# The Development of a Recombinant scFv Monoclonal Antibody Targeting Canine CD20 for Use in Comparative Medicine

**DOI:** 10.1371/journal.pone.0148366

**Published:** 2016-02-19

**Authors:** Saurabh Jain, Luca Aresu, Stefano Comazzi, Jianguo Shi, Erin Worrall, John Clayton, William Humphries, Sandra Hemmington, Paul Davis, Euan Murray, Asmare A. Limeneh, Kathryn Ball, Eva Ruckova, Petr Muller, Borek Vojtesek, Robin Fahraeus, David Argyle, Ted R. Hupp

**Affiliations:** 1 University of Edinburgh, Institute of Genetic and Molecular Medicine and School of Veterinary Medicine, Edinburgh, EH4 2XR, United Kingdom; 2 Dipartimento di Biomedicina Comparata e Alimentazione (BCA) Department of Comparative Biomedicine and Food Science, Università di Padova 35020 Legnaro (PD), Italy; 3 Dipartimento di Scienze Veterinarie e Sanità Pubblica, Università degli Studi di Milano, via Celoria 10, 20133 Milano, Italy; 4 Mologic, Ltd, Bedford Technology Park, Thurleigh, Bedford, MK44 2YP, United Kingdom; 5 Bahit Dar University College of Medicine and Health Sciences Department of Medical Biochemistry and Molecular Biology, Bahir Dar, Ethiopia; 6 Regional Centre for Applied Molecular Oncology, Masaryk Memorial Cancer Institute, 656 53 Brno, Czech Republic; 7 INSERM Unité 940, Institut de Génétique Moléculaire, Université Paris 7, Hôpital St Louis, 27 rue Juliette Dodu, Paris, France; LMU Munich, GERMANY

## Abstract

Monoclonal antibodies are leading agents for therapeutic treatment of human diseases, but are limited in use by the paucity of clinically relevant models for validation. Sporadic canine tumours mimic the features of some human equivalents. Developing canine immunotherapeutics can be an approach for modeling human disease responses. Rituximab is a pioneering agent used to treat human hematological malignancies. Biologic mimics that target canine CD20 are just being developed by the biotechnology industry. Towards a comparative canine-human model system, we have developed a novel anti-CD20 monoclonal antibody (NCD1.2) that binds both human and canine CD20. NCD1.2 has a sub-nanomolar K_d_ as defined by an octet red binding assay. Using FACS, NCD1.2 binds to clinically derived canine cells including B-cells in peripheral blood and in different histotypes of B-cell lymphoma. Immunohistochemical staining of canine tissues indicates that the NCD1.2 binds to membrane localized cells in Diffuse Large B-cell lymphoma, Marginal Zone Lymphoma, and other canine B-cell lymphomas. We cloned the heavy and light chains of NCD1.2 from hybridomas to determine whether active scaffolds can be acquired as future biologics tools. The V_H_ and V_L_ genes from the hybridomas were cloned using degenerate primers and packaged as single chains (scFv) into a phage-display library. Surprisingly, we identified two scFv (scFv-3 and scFv-7) isolated from the hybridoma with bioactivity towards CD20. The two scFv had identical V_H_ genes but different V_L_ genes and identical CDR3s, indicating that at least two light chain mRNAs are encoded by NCD1.2 hybridoma cells. Both scFv-3 and scFv-7 were cloned into mammalian vectors for secretion in CHO cells and the antibodies were bioactive towards recombinant CD20 protein or peptide. The scFv-3 and scFv-7 were cloned into an ADEPT-CPG2 bioconjugate vector where bioactivity was retained when expressed in bacterial systems. These data identify a recombinant anti-CD20 scFv that might form a useful tool for evaluation in bioconjugate-directed anti-CD20 immunotherapies in comparative medicine.

## Introduction

'One World Health' proposes the unification of human medical and veterinary sciences. This involves the establishment of collaborative ventures in clinical care, surveillance and control of cross-species disease, education, and research into disease pathogenesis, diagnosis, therapy and vaccination[1]. The concept encompasses the human population, domestic animals and wildlife, and the impact that environmental changes or 'environmental health' such as global warming will have on these populations[2,3]. Comparative medicine is the framework from which inter-species models can generate knowledge to impact on improved health of animal and humans in diseases linked to ageing such as cancer, infections like viruses, and even parasites that use animals as a vector for transmission to human populations[4].

Cancer remains one of the world’s leading causes of death in humans and the total economic impact of premature death and disability from cancer represents 1.5% of the world’s GDP, not including the direct cost of treatment or patient suffering[5]. The outstanding challenge in cancer research in the 21st century is the implementation of inter-disciplinary translation of rapidly growing advances in tumour biology into new anti-cancer treatment modalities as current preclinical models have not predicted high success rates in the clinic[6]. Rodent xenograft models are the current traditional preclinical test-bed and form an excellent model for initial basic pharmacologic research and safety investigations[7,8]. However, these artificial tumour models have a poor track record in predicting the clinical outcome and new pre-clinical models are urgently needed to translate advances in basic cancer research[9]. One approach would be the use of patient derived xenografts with a high degree of genetic similarity to the primary tumour to provide improved cancer models by implementing “personalized medicine”[10]. Additional innovative preclinical models are needed that are more predictive of human responses[11].

Comparative medicine within the one world health framework provides a platform for offering innovative cross-species models that inform on the complex biology of human diseases like cancer. The world-wide veterinary oncology field can offer compelling sporadic models of carcinogenesis in companion animals (cats and dogs) that mimic certain aspects of cancer development such as metastasis and immune-host cell interactions[12,13]. Spontaneous tumours in dogs share important clinical, pathological, immunologic, molecular, diagnostic, and therapeutic characteristics with corresponding human disease and may be treated with similar anti-cancer modalities as in humans[14,15]. As such, spontaneous canine cancers can provide a relevant model for some human cancers[16–19]. Companion therapeutics are thus emerging on the drug discovery landscape[20].

Over the past 30 years, a striking repertoire of recombinant monoclonal antibody scaffolds have been developed as novel therapeutics[21]. Some of the top-line treatments of human disease include the use of “humanized” monoclonal antibodies. Monoclonal antibodies therefore provide an untapped potential in fields that bridge diagnostics, synthetic biology, therapeutics, and immunology[22]. Towards realizing the potential of canine cancer as an innovative model for comparative immunotherapeutics, we focused on the proto-oncogenic CD20 receptor. This receptor falls in a subclass of cell surface differentiation antigens that are expressed in both normal and lymphoid cancer cells[23]. CD20 is a 33kDa tetraspanning transmembrane phosphoprotein which is expressed in pre B-cells but still the precise function of CD20 is unknown[24]. The human-mouse chimeric monoclonal antibody Rituximab targets the CD20 receptor in B-cell malignancies and has provided a compelling proof-of-concept that a recombinant monoclonal can be used as a biologic based drug treatment[25,26]. There is now growing evidence that Rituximab can affect other disease indications such as rheumatoid arthritis[27], immune related indications[28–30], and in transplantation[31].

Due to this widespread potential of CD20 targeting antibodies in disease indications, there are emerging Rituximab “biosimilars” for use in humans[32] such as Ibritumomab and Tositumomab[33–35]. Although the Rituximab and chemotherapy regime (R-CHOP) has a big impact on patient management, there are views towards ever improving anti-CD20 modalities. One solution to developing innovative immune therapies is the integration to sporadic canine Diffuse Large B-cell and Marginal Zone lymphomas that have a similar molecular fingerprint to the human counterpart[36,37]. Indeed, there is now reported an anti-CD20 mouse monoclonal antibody that can target canine CD20[38] and biopharmaceutical licensing of the FDA’s first canine-specific CD20 targeting monoclonal antibody[20]. Here we report on an approach to develop a recombinant version of a novel anti-CD20 monoclonal antibody that can target both canine and human CD20 protein and that can be cloned towards use as a biologics platform for recombinant anti-CD20 bioconjugates in comparative medicine. Although emerging trends in the pharmaceutical field are moving towards recombinant antibody based immunotherapies, there are a paucity of immunocompetent models existing with which to develop cutting-edge concepts using recombinant IgG. Key advantages of the recombinant scFV are (i) the ability to fully caninize the molecule or use synthetic constructs for repeat use *in vivo* through tolerance of the canine immune system and (ii) the development of ADCC based assays after fusion of the scFV to canine Fc constant chains.

## Materials and Methods

### Production of mouse anti-canine CD20 Antibody

**Fig 1:**
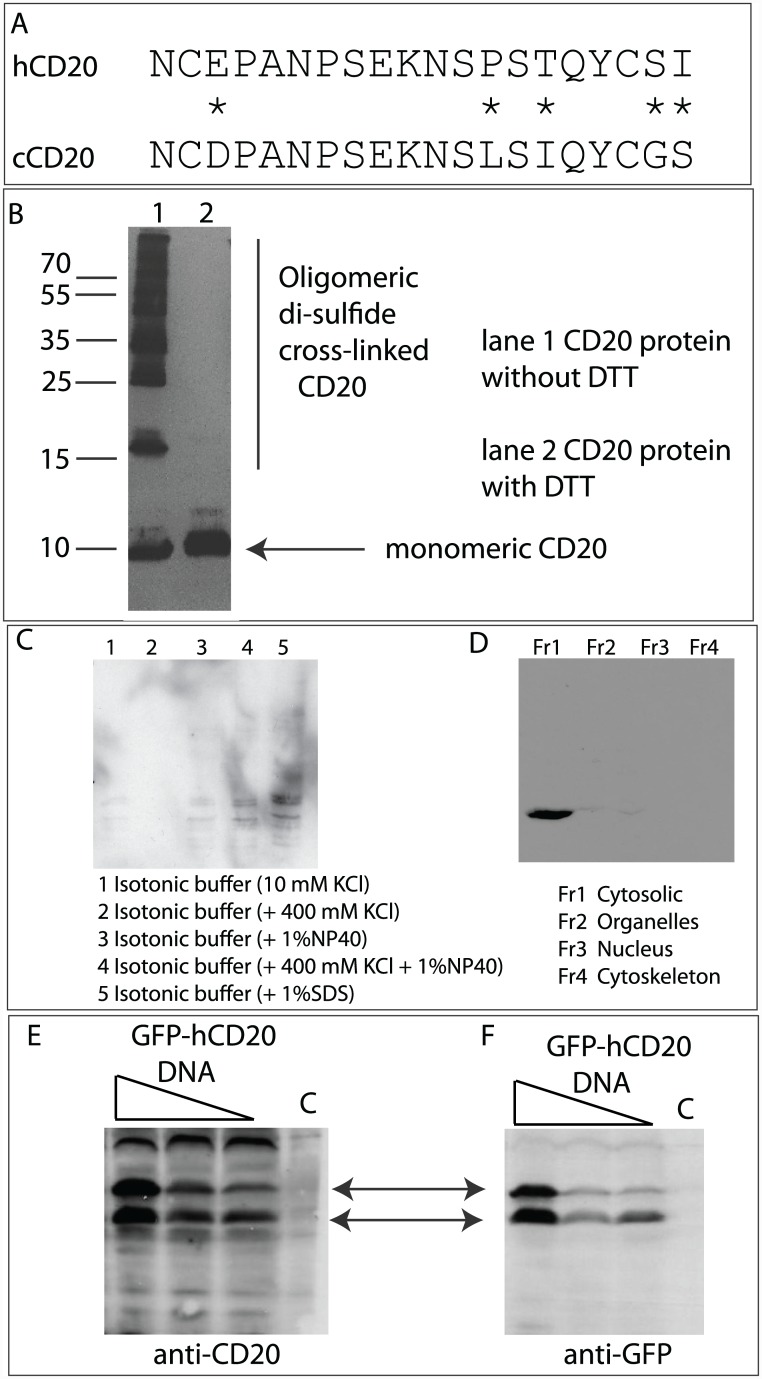
Isolation of a mouse CD20-specific monoclonal antibody that can recognize human and canine CD20 protein. *(A)*. *Amino acid sequence of human and canine CD20 surrounding the Rituximab epitope*. The asterisks highlight divergence between human (hCD20) and canine (cCD20) proteins. **(***B)*. *Immunoblotting of recombinant canine CD20 protein*. Bacterially expressed his-tagged canine CD20 protein (amino acids 140–190) was purified by nickel chromatography and after electrophoresis without and with DTT, immunoblotted with MAB NCD1.2. The laddering observed without DTT presumably reflects inter-molecular di-sulfide bonds through the two cysteine residues. *(C and D)*. *(C)*. *Immunoblotting of authentic canine CD20 protein*. 3132 canine lymphoma cells were lysed with different lysis buffers (as indicated) to determine optimal extraction buffer to immunoblot endogenous CD20 protein. (D). 3132 canine lymphoma cells were subjected to chemical fraction (Fr1-Fr4, as indicated) to isolate compartments to determine the dominant localization of CD20 protein in 3132 cells. (*E and F)*. *Immunoblotting of human CD20 protein*. Transfection of human GFP-CD20 into H1299 cells demonstrated that NCD1.2 can bind to human CD20 (Left panel) with and anti-GFP monoclonal antibody as a control for protein expression and relative molecular mass (right panel).

CHO-k1 and H1299 cells were obtained from ATCC. Balb/c mice aged 5–8 weeks were immunized by and purchased from the service provider Moravian Biotechnology Ltd (who contract immunization to the Veterinary Research Institute (University of Brno, Czech Republic) under the animal license number 14828/2010–17210 falling under European Union law) with a final concentration of 100 μg/ml canine CD20 peptide (NCDPANPSEKNSLSIQYCGS; Fig 1A) in complete Freud’s adjuvant and subsequent injections were performed with 25 μg/ml of CD20 peptide. Subsequently, tail bleed was obtained to determine the antibody titre and once a high titre response was reached, the mice were given a final boost into the peritoneal cavity and sacrificed 7 days later. Using these antibody producing spleen cells and myeloma cells, standard hybridoma technology was used in order to obtain hybridoma cells[39]. Hybridoma cell lines were obtained from Moravian Biotechnology, and the authors did not house any animals or conduct experiments on animals. Hybridomas producing IgG that recognized CD20 peptide as well as protein were sub-cloned twice by limiting dilution. Following the isolation and cloning of hybridomas, the binding of the secreted monoclonal antibodies was determined via several in vitro assays that are further discussed. The monoclonal antibody we characterize in this study is named NCD1.2. Hybridoma cell lines were obtained from Moravian and the research involved on animals housing or on experiments with animals.

### Octet binding antibody-peptide binding assay

The binding assay was carried out using streptavidin-coated biosensors on an Octet RED biolayer interferometry system (FortéBio Inc.) that measures binding to the sensor tip as a wavelength shift (in nm) in real time. All the steps of the assay process were performed at 30°C with the plate shaking speed set at 1000 rpm. 96-well microtitre plates (Greiner Bio) were made up using 200 μl volumes. Analytes were diluted with PBS (pH 7.4) containing 0.02% Tween 20, 0.005% sodium azide and 100 μg/mL bovine serum albumin (Kinetics Buffer—Forte Bio). Biotinylated CD20 peptide was loaded onto to 8 sensors from a 2 μg/mL solution containing 2 mM dithiothreitol (ex Sigma-Aldrich) until a wavelength shift of 0.5 nm had been achieved. The association data were collected for 10 minutes from solutions of monoclonal CD20 antibody where the concentration was varied between 32 nM and 0.5 nM by half serial dilution. The disssociation step was carried out over a 40 minute time period in Kinetics Buffer. The assay data were processed using Data Analysis (version 6.3 –Forte Bio) to obtain kinetic values. Briefly, a buffer blank was used as a reference cell subtraction and the data series was evaluated using a global fit algorithm for 1:1 binding interaction. Fluorescent polarization assay was performed using the anti-canine CD20 monoclonal antibody onto FITC tagged human CD20 peptide, with the human-mouse chimeric monoclonal antibody Rituximab as the control antibody. Purified monoclonal antibody NCD1.2 and Rituximab diluted in PBS were titrated against 30 nM fluorescein-labeled peptide corresponding to human CD20 (NCEPANPSEKNSPSTQYCYS) in assay buffer (PBS, 0.05% Tween-20). To subtract the nonspecific binding, we used purified mouse immunoglobulins with the same concentration as analyzed antibodies. All reactions were carried out in a total volume of 60 μl per well of a 96-well black Nunc Plate (Sigma-Aldrich). The plate was incubated for 1 hr at room temperature with shaking. Fluorescence polarization was measured at 21°C using FilterMax™ F5 Multimode Microplate Reader (Molecular Devices) with excitation and emission wavelengths of 485 nm and 535 nm, respectively.

### Transfection of human CD20 into H1299 cells

Primers introducing BamHI and XhoI sites at the ends of reported sequence (Genbank accession number NP_068769.2) of human CD20 were designed for its amplification and cloning into an EGFP-expression vector (N1). H1299 cells were grown as monolayers in RPMI 1640 supplemented with 10% fetal bovine serum (FBS). 1μg of DNA (GFP-CD20) was transfected in H1299 cells, along with GFP as a negative control using Attractene in order to measure the binding efficiency of the mouse anti-canine monoclonal antibody to its human CD20 counterpart. The binding was determined via Western blot as well as Immunofluorescence. Chemical fractionation into compartments was carried out using a fraction kit that separates into cytosolic, membrane/organelle, nuclear, and cytosolic/insoluble proteomes (Calbiochem).

### Immunohistochemistry and FACS

Canine samples were taken as part of normal diagnostic procedures from dogs treated by private veterinary practitioners for diagnostic and immunophenotyping analysis within veterinary schools (SC and LA) with newly diagnosed, previously untreated lymphoma. The samples are anonymized and unlinked and the authors have permission and authority to use the samples for research purposes. Biopsies were obtained from dogs with different types of B and T cell lymphomas, including Diffuse Large B-cell lymphoma (DLBCL), Marginal Zone Lymphoma (MZL), Follicular Lymphoma (FL), B-cell small lymphocytic (B-SLL), and Lymphoma and Peripheral T-cell Lymphoma (PTCL). All lymphoma samples were taken as part of normal diagnostic procedures from dogs with newly diagnosed, previously untreated lymphoma and the data are anonymized and unlinked[40]. For tissue micro array (TMA), hematoxylin and eosin-stained sections from each paraffin-embedded, formalin fixed block were used to define diagnostic areas and cores were obtained from each case and inserted in a grid pattern into a recipient paraffin block using a tissue arrayer. Sections were then cut and stained with antibodies to CD20 as well as CD79[41]. Following deparaffinization, heat induced antigen retrieval techniques were used for each antibody. Sections were analyzed for presence of positive immunolabelling to the mouse anti-canine CD20 antibody. Flow cytometry (FCM) expression of CD20 was evaluated on fine needle aspirates in RPMI 1640 medium (Invitrogen) obtained from dogs with different lymphoma subtypes. On each sample immunophenotyping was performed according to the previously published protocols in order to define immunophenotype and to identify the lymphoma subtype[42]. Evaluation of expression of membranous CD20 was performed using the following procedure: To avoid any non-specific binding, cells (adjusted at 5x 10^5^cells per tube) were incubated in RPMI 1640 medium containing 5% fetal bovine serum (FBS) and 0.2% sodium azide, followed by resuspension in PBS. One μl of mouse anti-canine CD20 Ab was added and incubated for 20 mins at 4°C. After couple of washes in RPMI/FCS/sodium azide, 2μl of goat anti-mouse Ig (FITC, Becton Dickinson) was added and incubated in the dark for 20 mins at 4°C. To evaluate non-specific binding of secondary antibody, one of the vials was used with lack of primary antibody. Isotype control was not used due to the lack of isotype matched available antibody. Following the incubation, cells were washed and cells were acquired using FacScalibur equipment (Becton Dickinson). The acquisition parameters were kept constant for the duration of the study and the machine was routinely calibrated using calibration beads (CaliBRITE, Becton Dickinson, San Jose, CA, USA) to ensure comparable readings between different days. A minimum of 10,000 events was gated to exclude dead cells, and analysis was performed with Cell Quest software (Becton Dickinson, San Jose, CA, USA). Thus, the efficiency of anti-canine CD20 antibody was determined on different types of B and T cell lymphomas that are meant to be positive and negative for CD20, respectively using FACS. For the competition binding of unlabeled Rituximab or NCD1.2 to the human SUDHL4 B-cell lymphoma cell line expressing CD20, incubations were with Rituximab conjugated by DyLight 488 and analzyed by FACS. The summary of the raw data is in Table 1.

**Table 1:** 

Sample	Events	Parent	Tube_001:P3% Grandparent	Tube_001:P3% Total	Mean	Geo Mean	SD	Median
Control Dylight-488-Rituximab 10 mg/l	20107	57.91	57.46	49.05	12426	10330	6883.65	11067
+ Rituximab 500 mg/l	20067	59.77	59.42	53.12	242	12	1883.81	19
+ Rituximab 200 mg/l	20060	67.86	67.51	59.95	77	34	668.5	48
+ Rituximab 100 mg/l	20126	68.58	68.23	60.61	129	101	178.37	111
+ Rituximab 50 mg/l	20021	65.61	65.21	56.56	1333	1062	875.13	1153
+ NCD 500 mg/l	20245	58.98	58.53	50.2	12209	10456	6734.99	10821
+ NCD 200 mg/l	20008	62.15	61.56	51.38	12340	10511	6753.15	11093
+ NCD 100 mg/l	20036	61.05	60.58	50.93	12254	10547	6628.63	10932
+ NCD 50 mg/l	20032	61.4	60.85	50.65	12823	11053	6925.6	11465

### Construction of mouse anti-canine CD20 scFv library from NCD1.2 hybridoma cells

RNA was extracted using Trizol from the NCD1.2 hybridoma cells and reverse transcription (Qiagen) was used to obtain cDNA. The set of primers used for amplification from cDNA of the variable chains and to splice both variable regions to make single chain variable fragments (scFv) are in Tables 2–6 [43]. The heavy and light chains (10 ng of each) were then annealed together using serine glycine linkers (60 pmol of each; below) to form ~800bp products (scFv) by splice overlap extension PCR^54^. The sequence of the RSC-F (sense) was 5′ GAG GAG GAG GAG GAG GAG GCG GGG CCC AGG CGG CCG AGC TC 3′ and that of RSC-B (reverse) was 5′ GAG GAG GAG GAG GAG GAG CCT GGC CGG CCT GGC CAC TAG TG 3′. The PCR products were gel extracted and pooled together, restriction digested with *SfiI* and ligated into *SfiI* digested pCOMB3xSS. Subsequently, the ligated product was transformed into electro competent cells via electroporation and the library was titered. 10 clones from the library were analysed by restriction digestion using *SfiI* to calculate the insert percentage.

**Table 2:** Vк chain 5’ primers: MSCVK 1–17 for construction of a murine scFv library (pCOMB3xSS).

MSCVK1	5′ GGG CCC AGG CGG CCG AGC TCG AYA TCC AGC TGA CTC AGC C 3′
MSCVK2	5′ GGG CCC AGG CGG CCG AGC TCG AYA TTG TTC TCW CCC AGT C 3
MSCVK3	5′ GGG CCC AGG CGG CCG AGC TCG AYA TTG TGM TMA CTC AGT C 3′
MSCVK4	5′ GGG CCC AGG CGG CCG AGC TCG AYA TTG TGY TRA CAC AGT C 3′
MSCVK5	5′ GGG CCC AGG CGG CCG AGC TCG AYA TTG TRA TGA CMC AGT C 3′
MSCVK6	5′ GGG CCC AGG CGG CCG AGC TCG AYA TTM AGA TRA MCC AGT C 3′
MSCVK7	5′ GGG CCC AGG CGG CCG AGC TCG AYA TTC AGA TGA YDC AGT C 3′
MSCVK8	5′ GGG CCC AGG CGG CCG AGC TCG AYA TYC AGA TGA CAC AGA C 3′
MSCVK9	5′ GGG CCC AGG CGG CCG AGC TCG AYA TTG TTC TCA WCC AGT C 3′
MSCVK10	5′ GGG CCC AGG CGG CCG AGC TCG AYA TTG WGC TSA CCC AAT C 3′
MSCVK11	5′ GGG CCC AGG CGG CCG AGC TCG AYA TTS TRA TGA CCC ART C 3′
MSCVK12	5′ GGG CCC AGG CGG CCG AGC TCG AYR TTK TGA TGA CCC ARA C 3′
MSCVK13	5′ GGG CCC AGG CGG CCG AGC TCG AYA TTG TGA TGA CBC AGK C 3′
MSCVK14	5′ GGG CCC AGG CGG CCG AGC TCG AYA TTG TGA TAA CYC AGG A 3′
MSCVK15	5′ GGG CCC AGG CGG CCG AGC TCG AYA TTG TGA TGA CCC AGW T 3′
MSCVK16	5′ GGG CCC AGG CGG CCG AGC TCG AYA TTG TGA TGA CAC AAC C 3′
MSCVK17	5′ GGG CCC AGG CGG CCG AGC TCG AYA TTT TGC TGA CTC AGT C 3′

**Table 3:** Vк chain 3’ primers for construction of a murine scFv library (pCOMB3xSS).

MSCJK12-BL	5′ GGA AGA TCT AGA GGA ACC ACC CCC ACC ACC GCC CGA GCC ACC GCC ACC AGA GGA TTT KAT TTC CAG YTT GGT CCC 3′
MSCJK4-BL	5′ GGA AGA TCT AGA GGA ACC ACC CCC ACC ACC GCC CGA GCC ACC GCC ACC AGA GGA TTT TAT TTC CAA CTT TGT CCC 3′
MSCJK5-BL	5′ GGA AGA TCT AGA GGA ACC ACC CCC ACC ACC GCC CGA GCC ACC GCC ACC AGA GGA TTT CAG CTC CAG CTT GGT CCC 3′

**Table 4:** Vλ 5’ and 3’ primers for construction of a murine scFv library (pCOMB3xSS).

V_λ_5’ forward Primer MSCVL-1	5′ GGG CCC AGG CGG CCG AGC TCG ATG CTG TTG TGA CTC AGG AAT C 3′
V λ 3’ Reverse Primer MSCJL-BL	5′ GGA AGA TCT AGA GGA ACC ACC CCC ACC ACC GCC CGA GCC ACC GCC ACC AGA GGA GCC TAG GAC AGT CAG TTT GG 3′

**Table 5:** V_H_ 5' Sense primers: MSCVH1-MSCVH19 for construction of a murine scFv library (pCOMB3xSS).

MSCVH1	5′ GGT GGT TCC TCT AGA TCT TCC CTC GAG GTR MAG CTT CAG GAG TC 3′
MSCVH2	5′ GGT GGT TCC TCT AGA TCT TCC CTC GAG GTB CAG CTB CAG CAG TC 3′
MSCVH3	5′ GGT GGT TCC TCT AGA TCT TCC CTC GAG GTG CAG CTG AAG SAS TC 3′
MSCVH4	5′ GGT GGT TCC TCT AGA TCT TCC CTC GAG GTC CAR CTG CAA CAR TC 3′
MSCVH5	5′ GGT GGT TCC TCT AGA TCT TCC CTC GAG GTY CAG CTB CAG CAR TC 3′
MSCVH6	5′ GGT GGT TCC TCT AGA TCT TCC CTC GAG GTY CAR CTG CAG CAG TC 3′
MSCVH7	5′ GGT GGT TCC TCT AGA TCT TCC CTC GAG GTC CAC GTG AAG CAG TC 3′
MSCVH8	5′ GGT GGT TCC TCT AGA TCT TCC CTC GAG GTG AAS STG GTG GAA TC 3′
MSCVH9	5′ GGT GGT TCC TCT AGA TCT TCC CTC GAG GTG AWG YTG GTG GAG TC 3′
MSCVH10	5′ GGT GGT TCC TCT AGA TCT TCC CTC GAG GTG CAG SKG GTG GAG TC 3′
MSCVH11	5′ GGT GGT TCC TCT AGA TCT TCC CTC GAG GTG CAM CTG GTG GAG TC 3′
MSCVH12	5′ GGT GGT TCC TCT AGA TCT TCC CTC GAG GTG AAG CTG ATG GAR TC 3′
MSCVH13	5′ GGT GGT TCC TCT AGA TCT TCC CTC GAG GTG CAR CTT GTT GAG TC 3′
MSCVH14	5′ GGT GGT TCC TCT AGA TCT TCC CTC GAG GTR AAG CTT CTC GAG TC 3′
MSCVH15	5′ GGT GGT TCC TCT AGA TCT TCC CTC GAG GTG AAR STT GAG GAG TC 3′
MSCVH16	5′ GGT GGT TCC TCT AGA TCT TCC CTC GAG GTT ACT CTR AAA GWG TST G 3′
MSCVH17	5′ GGT GGT TCC TCT AGA TCT TCC CTC GAG GTC CAA CTV CAG CAR CC 3′
MSCVH18	5′ GGT GGT TCC TCT AGA TCT TCC CTC GAG GTG AAC TTG GAA GTG TC 3′
MSCVH19	5′ GGT GGT TCC TCT AGA TCT TCC CTC GAG GTG AAG GTC ATC GAG TC 3′

**Table 6:** V_H_ 3’ primers for construction of a murine scFv library (pCOMB3xSS).

MSCG1ab-B	5′ CCT GGC CGG CCT GGC CAC TAG TGA CAG ATG GGG STG TYG TTT TGG C 3′
MSCG3-B	5′ CCT GGC CGG CCT GGC CAC TAG TGA CAG ATG GGG CTG TTG TTG T 3′
MSCM-B	5′ CCT GGC CGG CCT GGC CAC TAG TGA CAT TTG GGA AGG ACT GAC TCT C 3′

### Biopanning using the NCD1.2:scFv phage library

Phage preparation: Phage displaying scFv library was grown and rescued overnight at 30°C using helper phage M13KO7. The overnight phage library was centrifuged at 9000rpm for 20 mins. The phage supernatant was transferred to fresh tubes and 2g of PEG (4%) along with 1.5g NaCl (3%) were added. This mixture was incubated at 37°C shaking at 220rpm until fully dissolved. The cultures were again spun down at 9000rpm for 20 mins at 4°C and dry inverted for 10 minutes. This input phage was then added to the immunotube (pre-coated with 1 μg/ml streptavidin in water overnight at 4°C) which was coated overnight with biotinylated CD20 peptide (biotin-SGSGNCDPANPSEKNSLSIQYCGS) for each round and after incubating for 1 hour the immunotube was washed several times with 0.1% (v/v) PBS-T. The bound phage was eluted using 10 μg/ml Trypsin and re-infected into TG1 cells after they reached an OD of 0.4 and re-infected cells were grown for successive round of biopanning. The amount of washes was increased in subsequent rounds. Four rounds of panning were performed and each of the phage pools was stored at 4°C until used. The efficiency of the library was determined using the phage pools via ELISA. The ELISA plates were incubated with streptavidin and the protocol (above) was followed with different dilutions of the phage pools as the primary antibody and HRP conjugated anti-M13 was used as the secondary antibody. Subsequently, phage pools were titered onto LB (100 μg/ml Ampicillin) plates and the colonies were grown from round 3 and 4 in 200μl LB (100 μg/ml Ampicillin), expression of the antibody was induced with 1mM IPTG and secreted antibody was tested by monoclonal ELISA using HRP conjugated anti-protein A antibody. The positive clones were checked for stability, cloned into pCDNA3.1 and pTRC-His (B) for mammalian and bacterial expression respectively.

### Mammalian and Bacterial expression of scFv-3 and scFv-7

scFv-3 and scFv-7 were cloned into the *KpnI-EcoRI* sites in the bacterial and mammalian expression vectors, pTRCHis(B) and pCDNA3.1 (Clontech), respectively. The mammalian expression was carried out by transfection of CHO-k1 cells with the indicated scFv expression vector using Attractene Reagent. A day before transfection, CHO cells were split in 3 wells of a 6-well plate at density of 1 x 10^4^, 5 x 10^4^, and 1 x 10^5^, respectively. Next day, for each well, 0.2 μg of construct was mixed with 60 μl medium (without serum) first, then 1.5 μl of Attractene Reagent (Qiagen) was added in the above solution. The solutions were incubated at RT for 15 min to allow the complex formation, and then the transfection complexes was added drop-wise onto the cells in 2 ml of fresh media. The cells from each well were split into 100 mm tissue culture dishes the following day in 10 ml selective medium to allow individual colony formation. Finally, the individual colonies were transferred into 24-well plate (one colony per well, 96 colonies were picked up for each antibody). After 4 rounds of selection by ELISA, the best colonies (higher yield and low cell density) were cultured at a large scale in selective media (DMEM supplemented with 10% low IgG FBS (Life Technologies Ltd, USA), 1% Penicillin/Streptomycin, 2% L-glutamine, 250 μg/ml Mycophenolic acid (Sigma Aldrich, UK), 12.5 μg/ml Xanthine (Sigma Aldrich, UK), 200 ng/ml Geneticin (Invitrogen Ltd, UK)). The bacterial expression of scFv-3 and scFv-7 was carried out in BL21 after induction of expression using IPTG. The resuspended pellets were thawed on ice and resuspended in 10ml lysis buffer (50mM NaH_2_PO4, 300mM NaCl, 10mM imidazole, pH 8.0). Lysozyme was directly added at a final concentration of 100 μg/ml and the solution was stirred on ice for 30 minutes. Cells were sonicated on ice for 7 x 12 seconds with a 15 second interval between each cycle and the crude lysate was centrifuged for 30 minutes at 10,000g at 4°C to remove cellular debris. The supernatant was transferred to a clean tube for subsequent purification.

### Purification of His-tagged scFv and scFv-CPG2 bioconjugates via metal affinity chromatography

The E. coli lysate was added to the NTA magnetic agarose beads (QIAGEN) in a 6ml column and equilibrated with 15ml equilibration column buffer (50mM NaH2PO4, 300mM NaCl, 10mM imidazole, pH 8.0) and incubated overnight rotating at 4°C. The following day, the column was allowed to empty by gravity flow and the flow-through was collected in a 15ml Falcon tube. The column was then washed with 100ml of washing buffer (50mM NaH2PO4, 300mM NaCl, 20mM imidazole, pH 8.0) to remove any non-specifically bound proteins. After washing, 4ml of elution buffer (50mM NaH2PO4, 300mM NaCl, 250mM imidazole, pH 8.0) was added to the column and incubated at 4°C for 1 hour. Following this incubation step, the sample was collected at room temperature. The efficiency of the purification process was evaluated using SDS-PAGE and Western Blot. The eluted antibody was buffer exchanged into PBS via dialysis. The concentration of the resultant antibody was determined using a NanoDrop 1000 spectrophotometer and then the solution was stored at -20°C. For Cloning of scFv-3 and scFv-7 into CPG2 fusions; first, CPG2 was cloned into the *NcoI-XhoI* linker region of pEHISTEV bacterial expression vector to create pJGS101; second, the scFv-3/7 was subcloned into the *SalI-NotI* site of pJGS101 to create pJGS201. The CPG2 was previously been used as a bioconjugate to a scFv targeting CEA[44] and was provided by Mologics (Bedford, UK). The scFv-3/7 fusion proteins were highly insoluble using the scFv lysis buffer (above) but soluble protein could be recovered in lysis buffer containing 100 mM Tris (pH 8.0), 500 mm NaCl, 10% Glycerol, 1 mM PMSF, 100 μg/ml lysozyme, 1 mM DTT, 2 mM MgCl_2_, and benzoase. Protein was eluted in imidazole buffer from the nickel affinity column in stabilization buffer containing 50mM Tris pH 8, 500mM NaCl, 10% glycerol, 1 M betain, 0,5% Triton x-100, and 250mM imidazole).

## Results

### Production of mouse anti-canine CD20 monoclonal antibody from murine hybridomas

Hybridomas were acquired after immunization with a peptide derived from canine CD20 (NCDPANPSEKNSLSIQYCGS; Fig 1A) that comprises the minimal epitope bound by Rituximab[45]. This canine sequence diverges by five amino acids from the human sequence (Fig 1A). A hybridoma cell producing an IgG monoclonal (named NCD1.2) that recognized recombinant bacterially expressed canine CD20 protein (amino acids 140–190 comprising the extracellular domain) (Fig 1B) was sub-cloned twice by limiting dilution to obtain pure hybridoma population (data not shown). The monoclonal antibody NCD1.2 was able to recognize endogenous canine CD20 protein in canine 3132 lymphoma cells (Fig 1C and 1D). Transfected human GFP-CD20 expression construct in H1299 cells also resulted in evidence of NCD1.2 cross-binding to the human orthologue (Fig 1E and 1F). The cross reaction with human and canine protein could permit cross-species therapeutic model developments of CD20 positive diseases in future.

**Fig 2:**
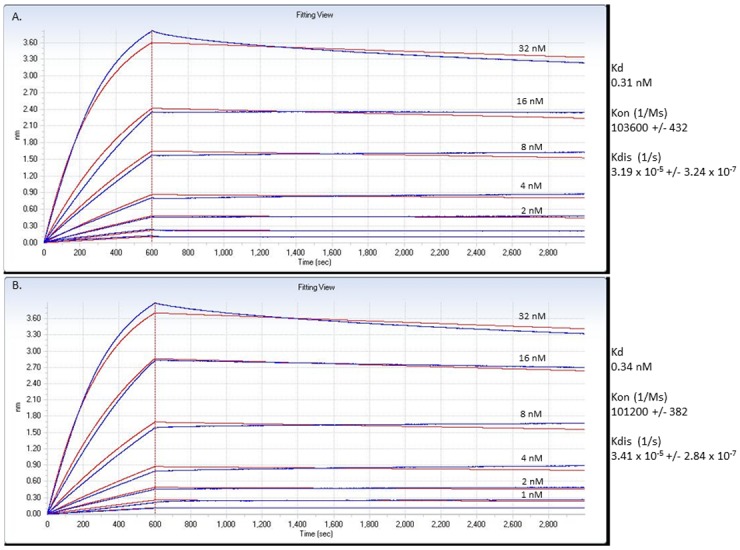
Definition of the relative affinity of the NCD1.2 MAB towards the epitope peptide. (A and B). NCD1.2 was titrated into reactions containing canine CD20 peptide on the solid phase in the absence or presence of DTT to evaluate potential oxidation effects on epitope binding. An Octet RED biolayer interferometry system that measures binding to the sensor tip as a wavelength shift (in nm) in real time. The assay data were processed using Data Analysis (version 6.3—Forte Bio) to obtain kinetic values as in the materials and methods and tabulated as K_d_, K_on_ and K_diss_.

**Fig 3:**
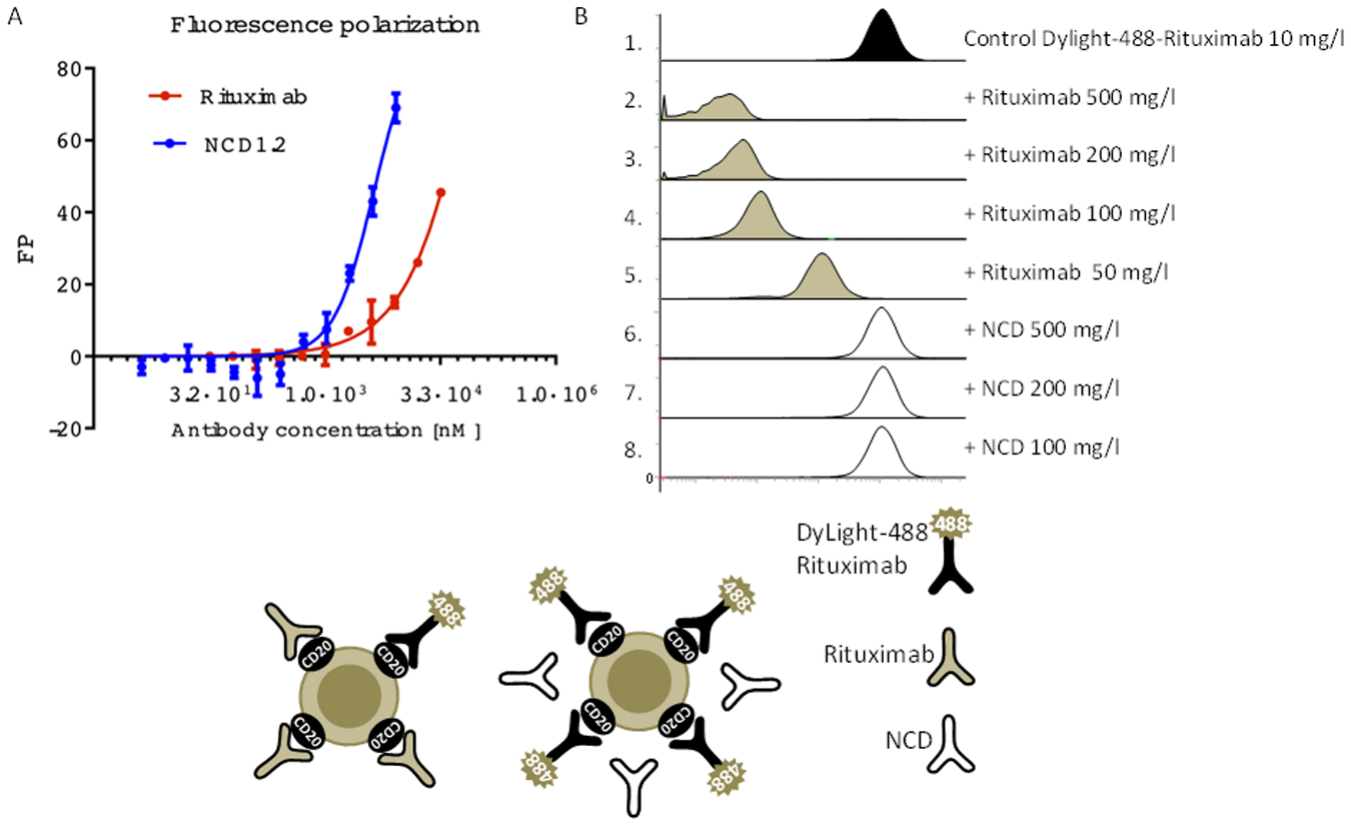
Comparison of NCD1.2 and Rituximab. (A). NCD1.2 and Rituximab were titrated into reactions containing fixed amounts of fluorescent human CD20 peptide (FITC; 30 nm peptide) and relative binding affinities were compared to each other in order to determine how the epitopes for the two monoclonal antibodies might differ. The data are plotted as changes in polarization as a function of increasing antibody concentration. (B). The human SUDHL4 B-cell lymphoma cell line expressing CD20 was incubated with Rituximab conjugated by DyLight 488 (10 μg/ml). The non-labeled Rituximab was added in increasing concentrations to samples 2–5. The non-labeled monoclonal antibody NCD1.2 was added to samples 6–8. The intensity of Rituximab-DyLight 488 was measured using flow cytometry.

We evaluated the relative affinity of the monoclonal antibody NCD1.2 to determine whether it might have use as a potential diagnostic or therapeutic in future. The Octet-Red system was used for measuring the relative on and off rates of the antibody to a biotinylated canine CD20 peptide on the solid phase. The relative K_d_ was defined to be 340 pM (Fig 2A). In inclusion of DTT in the reaction, which might disrupt potential intra or inter-molecular di-sulfide bonds in the CD20 epitope (Fig 1A) had no effects on the binding reaction with a K_d_ of 310 pM (Fig 2B). We cannot rule out the possibility that complex oxidation of the full-length CD20 protein (as in Fig 1B) might affect the epitope exposure on the receptor in live cells. A fluorescent (FITC) version of the human CD20 peptide sequence was used in fluorescence polarization to determine the relative affinity of the NCD1.2 MAb compared to Rituximab (Fig 3A). Purified monoclonal antibody NCD1.2 and Rituximab diluted in PBS were titrated against 30 nM fluorescein-labeled peptide corresponding to human CD20 (ncepanpseknspstqycys) in assay buffer (PBS, 0.05% Tween-20). These data confirm that the NCD1.2 MAB binds to both the human and canine sequences. Rituximab was used as a control, however, there was no detectable binding in this assay relative to NCD1.2, presumably as the Rituximab epitope differs form the canine sequence of CD20 and the MAB is reported to bind to a complex epitope on human CD20[45,46]. We also performed a converse competition assay on human cells expressing human CD20; we show that unlabeled Rituximab can compete with labeled Rituximab in binding to human cells; whereas the NCD1.2 does not compete for binding (Fig 3B and Table 1). These data indicate that NCD1.2 appears to have a different epitope than Rituximab since NCD1.2: (i) binds to canine and human CD20 peptide (unlike Rituximab); (ii) NCD1.2 binds to a small peptide sequence derived from human CD20 (unlike Rituximab); and (iii) Rituximab binds to human CD20+ cancer cells under conditions in which NCD1.2 is unable to compete.

### Reactivity of NCD1.2 to canine CD20 protein from clinical samples

**Fig 4:**
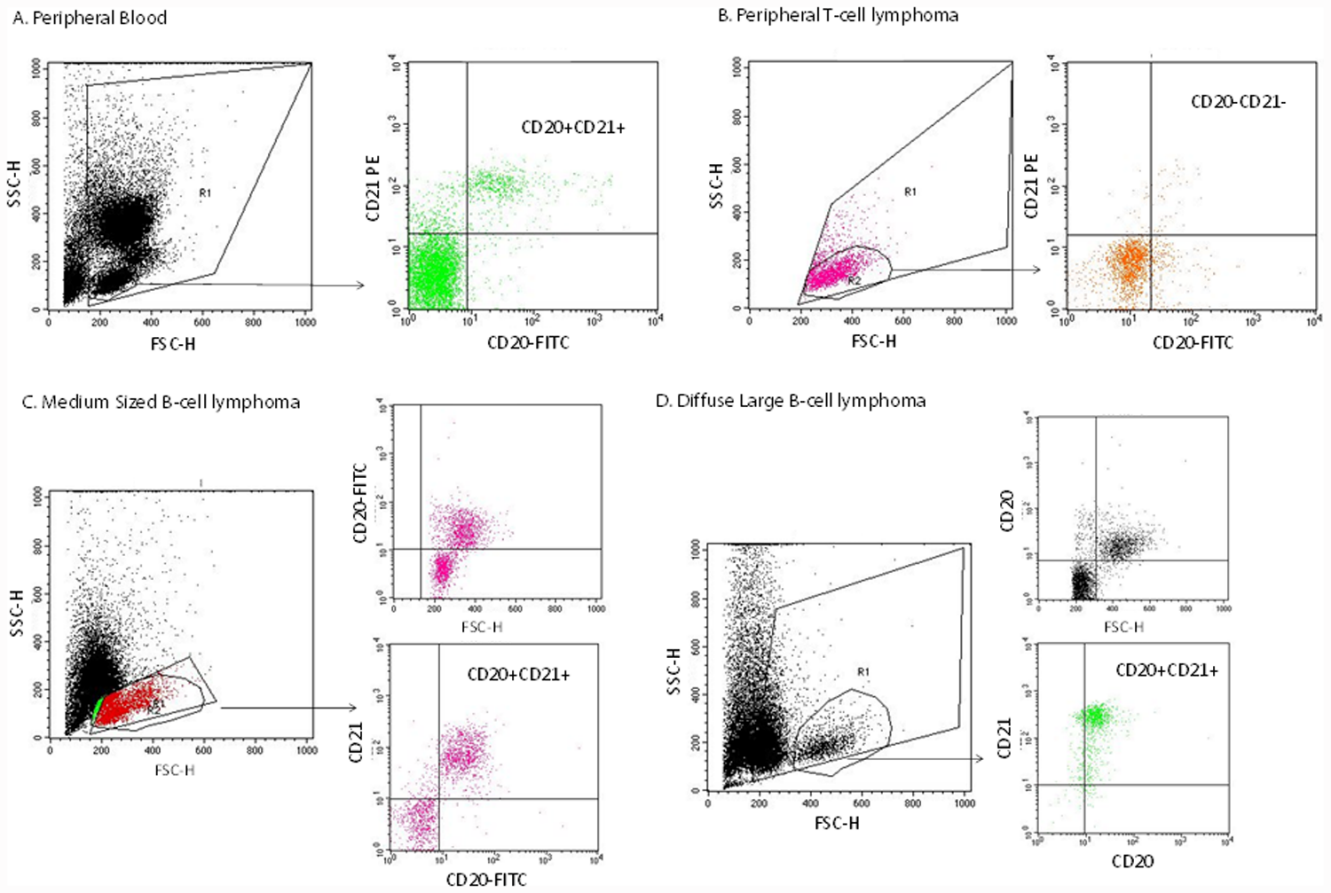
Expression of CD20 protein in clinically-derived canine cell populations. The highlighted cell sample populations were isolated and processed as indicated in the materials and methods. Expression of CD20 (and CD21) in the population of cells were analyzed by FACS and include (A) peripheral blood; (B) peripheral T-cell lymphoma; (C) Medium sized B-cell lymphoma; and (D) Diffuse Large B-cell lymphoma.

**Fig 5:**
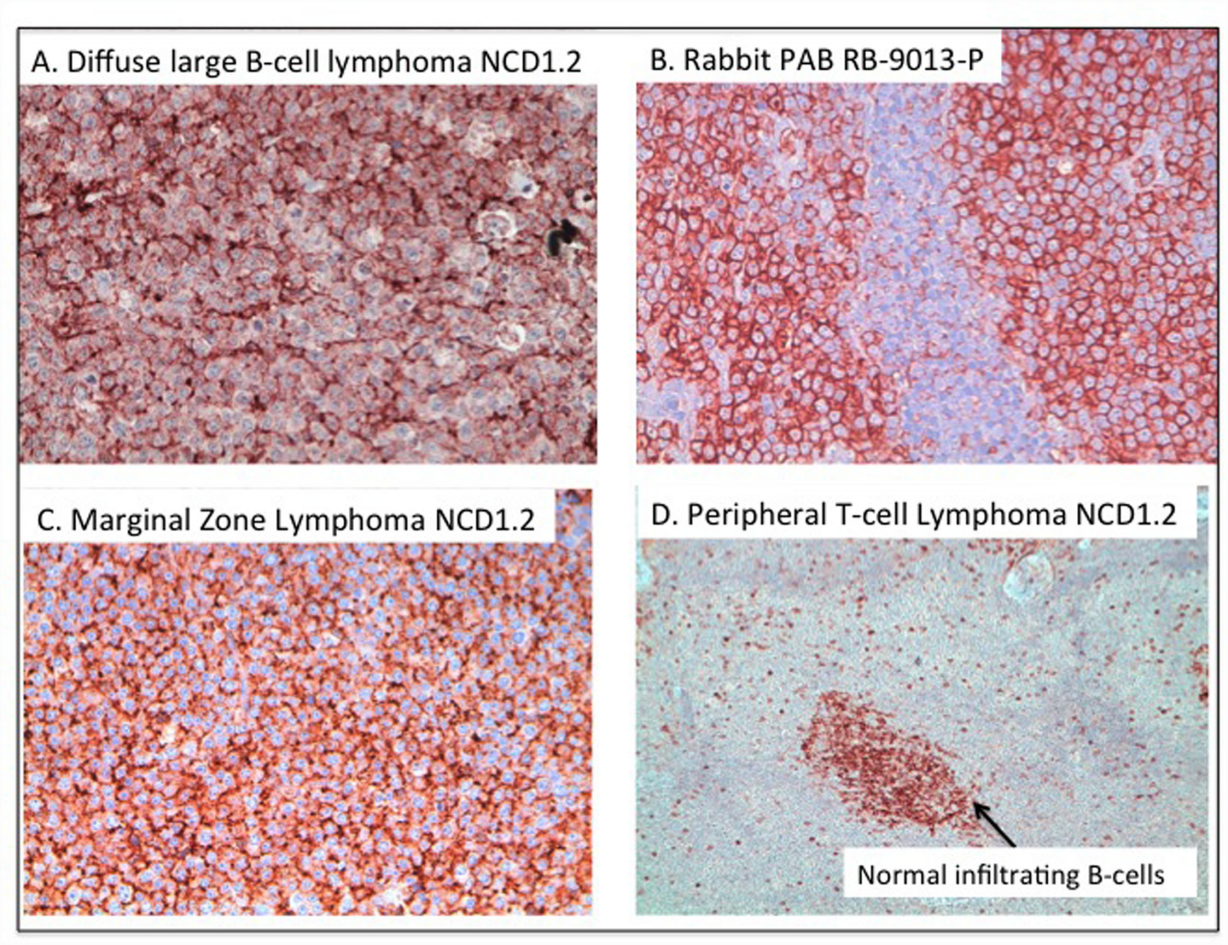
Expression of CD20 protein in formalin fixed paraffin imbedded canine cancer tissue. The indicated formalin-fixed, paraffin-embedded tissues were processed using immunohistochemistry as indicated in the materials and methods; and include representative images: (A) NCD1.2 staining in diffuse large B cell lymphoma (20x); (B) polyclonal anti-cd20 rabbit antibody staining in DLBCL (20x); (C) NCD1.2 staining in marginal zone lymphoma (20x); and (D) lack of NCD1.2 staining in peripheral T-cell lymphoma (10x); with infiltrating normal B-cells that are CD20+ highlighted by the arrow. The staining in brown highlights the position of the NCD1.2 reactive protein with nuclei stained in blue.

The CD20 antibody was evaluated using lymph nodal fine needle aspirates and blood samples preserved in RPMI 1640 medium from dogs with different lymphoma subtypes using flow cytometry. On each sample complete immunophenotyping was performed according to previously published procedures[41,42] in order to define cell origin and to identify the lymphoma subtype. These data reveal that NCD1.2 MAB can detect CD20-positive (CD21-positive) canine cells in normal peripheral blood (Fig 4A). Additionally, NCD1.2 was appropriately negative for detection in peripheral T-cell lymphoma (a CD20-negative cancer; Fig 4B), but could detect the antigen in medium-sized B-cell lymphoma (Fig 4C) or Diffuse Large B-Cell Lymphoma (Fig 4D). Following this, we also examined the expression of CD20 using NCD1.2 on formalin fixed samples which revealed diffuse membrane staining in Diffuse Large B-cell lymphomas (Fig 5A) similar to the polyclonal antibody used for clinical diagnosis (Fig 5B). Expression of CD20 protein was also observed as expected in a range of other lymphoma histotypes of B-cell origin, including Marginal Zone Lymphoma (Fig 5C), follicular lymphoma (data not shown), and small lymphocytic lymphoma (data not shown). The expected absence of CD20 protein in peripheral T-cell lymphoma (Fig 5D) was consistent with the FACS data (Fig 4D). Together, this validation of the monoclonal antibody NCD1.2, including its binding to canine and human CD20 protein and its binding to CD20 in the correct clinical tissue, prompted us to clone the antibody variable regions to determine whether it would be active as a recombinant antibody.

### Developing a scFv antibody phage library from hybridoma cell NCD1.2

**Fig 6:**
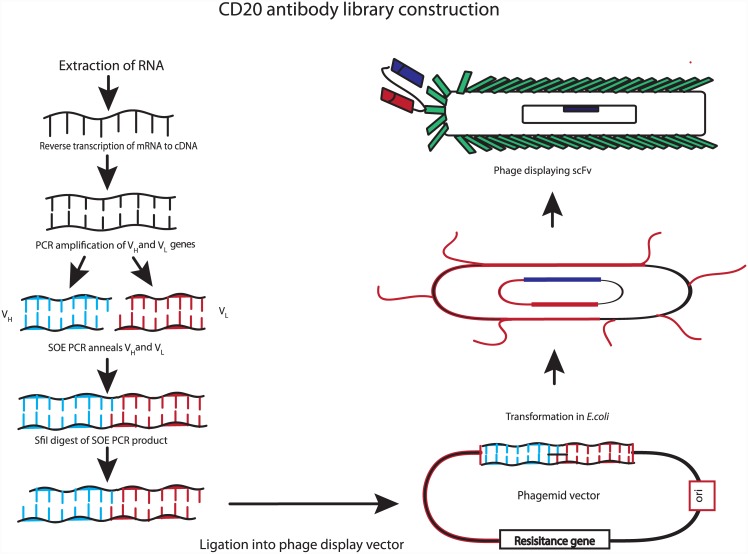
Strategy for cloning the variable light and heavy chains from the NCD1.2 hybridoma cell to create a NCD1.2 scFv-phage display library. RNA is extracted from hybridoma cell lines, followed by cDNA production. The cDNA was used as a template for the PCR-amplification of heavy and light chain variable regions using a diverse template primer set in order to capture the expressed sequences. The purified light and heavy chains were spliced together using PCR to create a 800 bp overlap product. This product was digested with *SfiI*, ligated into pCOMB3xSS, and transformed into TG1 cells. These cells are then be used to propagate the scFv-phage library for isolation of bioactive scFv-gIII fusion proteins.

**Fig 7:**
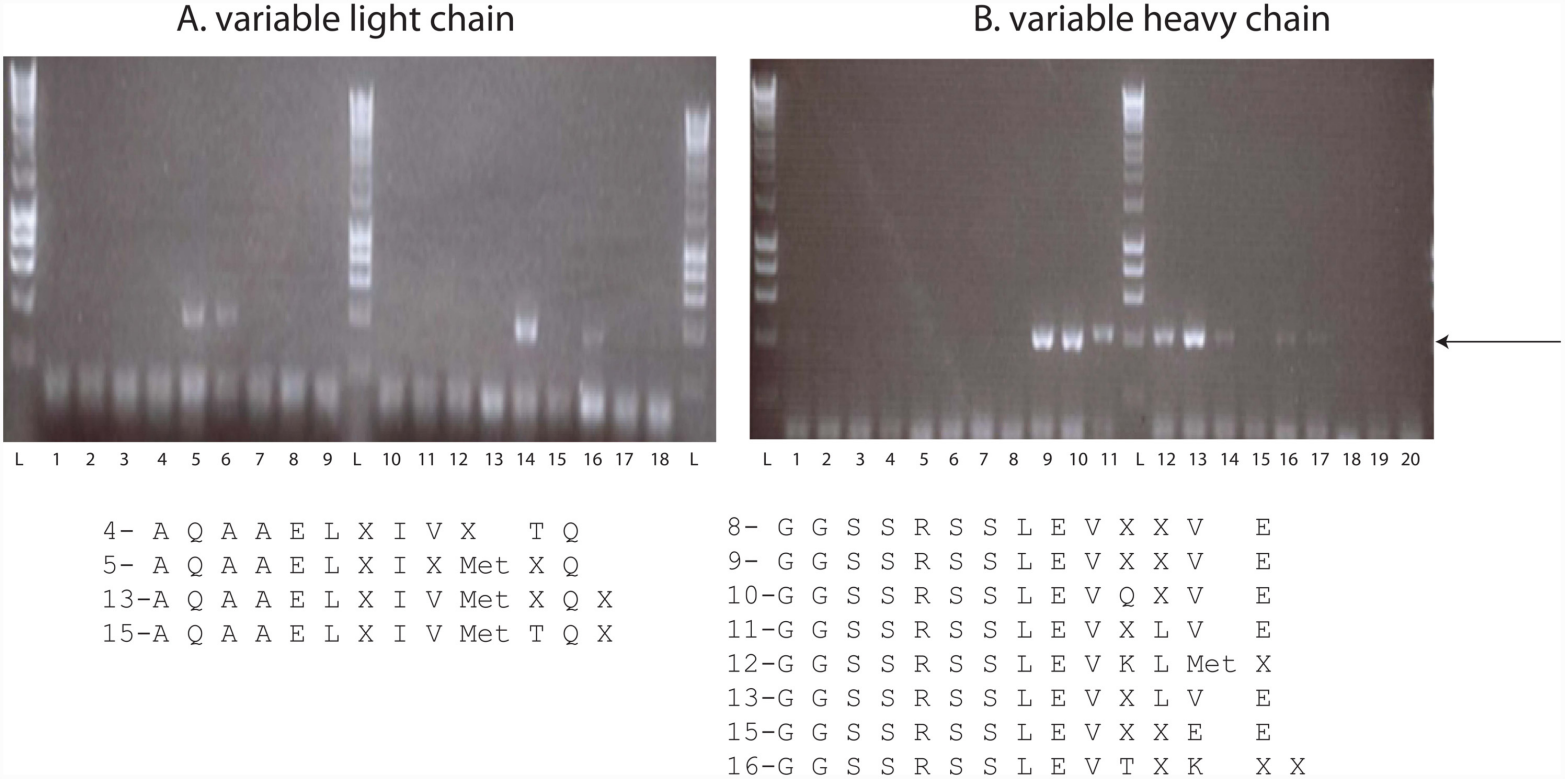
Amplification of heavy and light chains using PCR primer sets. RNA was isolated from NCD1.2 hybridoma cells and PCR amplified products (using the numbered oligonucleotide primers sets below each fig (decoded in the materials and methods) from cDNA were isolated on a 1.5% agarose gel: (A). Variable light chain amplification with 5’ sense and 3’ reverse primer sets: Lane L, ladder; Lane 1, negative control; Lanes 2–9, incorporate 5’ primers MSCVK1-MSCVK8; lanes 10–18 incorporate 5’ primers MSCVK9-MSCVK17. The 3’ primer reverse sets in each reaction were MSCJK12-BL, MSCJK4-BL, and MSCJK5-BL. (B). Variable heavy chain amplification with 5’ sense and 3’ reverse primer sets: Lane L, ladder; Lane 1, negative control; Lanes 2–11, incorporate 5’ primers MSCVH1-MSCVH10 and lanes 12–20 incorporate 5’ primers MSCVH11-MSCVH19. The 3’ primer reverse sets in each reaction were MSCM-B MSCG3-B MSCG1ab-B. The arrow highlights the position of the variable heavy or light gene. The amino acids sequences below each gel (from primers 4, 5, 13, and 15 for the light chain and primers 8, 9–13, 15, and 16 for the heavy chain) highlight the possible sequences of the framework regions to be expected in the sequences of the isolated, bioactive scFv.

The general strategy for cloning the heavy and light chain from the hybridoma secreting NCD1.2 is shown in Fig 6. Our approach was to clone and assemble the heavy and light chains into a single chain (scFv) to determine whether there would be any future potential of the recombinant biologic as a tool for imaging or therapeutics using bioconjugated synthetic biology approaches. As such as key question is whether or not scFv derived from NCD1.2 is bioactive as a single chain fused to gIII protein on the surface on M13 bacteriophage. Multiple PCR reactions were used with a diverse set of primers described previously[43] to convert cDNA from the mouse hybridoma NCD1.2 cell line into heavy chain (V_H_) and light chains (V_L_) DNAs. An example of the primer sets that could capture heavy and light chain PCR fragments is shown in Fig 7A and 7B. The PCR products from the V_H_ (primers 4, 5, 13, and 15) and V_L_ genes (primers 8, 9, 10, 11, 12, 13, 15, and 16) included a linker motif (SSRSSGG) from the PCR primers allowing a fusion of the single genes into a scFv by overlap extension PCR. The linker bridging the heavy and light chains is composed of a 23 amino acid glycine rich motif with the potential to stabilize ScFv dimers into diabodies that improve avidities[47]. The termini of the scFv PCR product were restricted using *SfiI* to clone into pComb3xSS for packaging into M13 bacteriophage (Fig 6).

### Isolation of bioactive recombinant antibodies from the scFv:NCD1.2 phage display library

**Fig 8:**
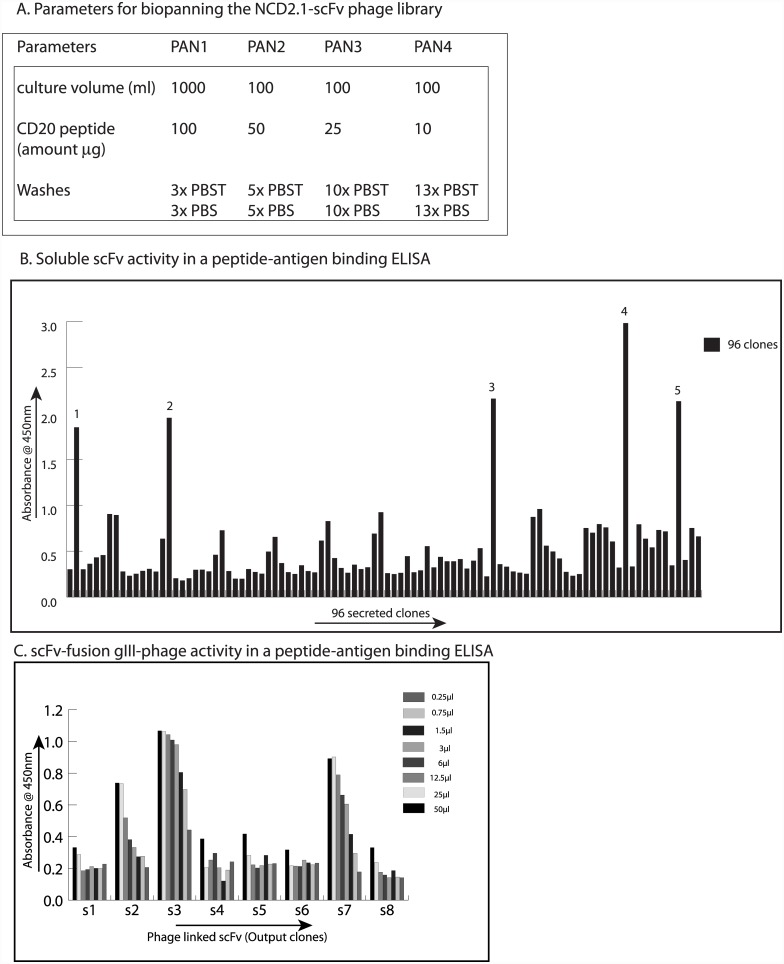
Isolation of bioactive recombinant scFv targeting CD20. (A). Soluble scFv fragment binding to its antigen. Biotinylated CD20 peptide (0.1 μg/ml in 50 μl) was coated onto streptavidin (1 μg/ml in 50 μl) coated solid phase and used as an antigen in the selection of scFv phage that bound to the CD20 epitope as indicated in the materials and methods. As the selection in rounds progressed, there was a reduction in the amount of antigen in the selection and an increased stringency of washing. (B). Colonies (96) from rounds 3 and 4 were grown in 200 μl of LB media and induced overnight at 30°C to overproduce soluble scFv fragments. Following a freeze-thaw and sonication protocol to release scFv from the bacterial debris, the clarified lysate was assayed in the ELISA with peroxidase conjugated to protein A. The binding was quantified using TMB and was measured in optical density at 450nm. Five high affinity scFv were identified (highlighted 1–5). However, their instability precluded their routine use (data not shown). (C). Phage producing plaques from rounds 3 and 4 were grown overnight, the scFv-phage in the supernatant was PEG precipitated, and assayed in an ELISA using biotinylated CD20 peptide. Active scFv-gIII-fusion phage was detected using an M13-phage antibody and binding was quantified using TMB and plotted as optical density (450 nm). Eight representative scFv-gIII fusion phage are highlighted where two bioactive recombinant phage (3 and 7) were isolated.

The scFv:NCD1.2-phage library was used in a biopanning screen through four rounds of selection using biotinylated-canine CD20 peptide. Colonies from rounds 3 and 4 (Fig 8A) were subjected to lysis to obtain soluble scFv protein unfused to gIII M13 coat protein to determine whether the minimal scFv fragment exhibits bioactivity. Approximately five high affinity scFv were obtained by this protocol (Fig 8B). These data indicate that the scFv:NCD1.2 phage library has bioactive antibodies that can be isolated.

The scFv-gIII phage protein has an amber codon in between the scFv and gIII M13 coat protein so that soluble scFv can be obtained when the phage is infected into a bacterial F-pillus host without an amber suppressor like HB2151. However, we found that this scFv isolated in soluble form was not stable for longer than 24 hours at 4°C. This instability of the purified scFv precluded its routine use (data not shown). As such, the scFv:NCD1.2 phage library was next subjected to a biopanning screen in which the activity was tested when the scFv fusion to the gIII protein remained intact. A representative ELISA is shown where two high affinity scFv:gIII:phage fusions were identified and were very stable (Fig 8C). The fusion to gIII protein may provide a stabilizing scaffold to the scFV much as a constant domain could in a full-length IgG.

**Fig 9:**
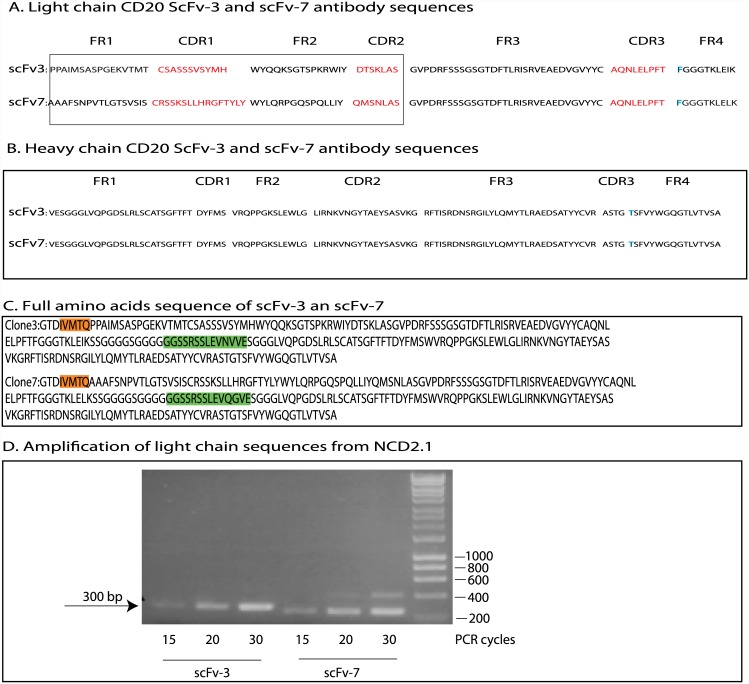
Amino acid sequences of scFv-3 and scFv-7. (A and B). The amino acid sequences of the light chain and heavy chain framework and CDR regions are as indicated. The CDRs of the light chain are highlighted in red, since the sequences diverge in CDR1 and CDR2 of the two light chains, but are identical in the CDR3 of the light chain. (C). The full amino acid sequence of scFv-3 and scFv-7 including the linker is highlighted. Based on the amino acid sequences, the scFv-3 and scFv-7 light chains could have been amplified using any of the primers 4,5,13, or 15 (Fig 7A (in orange)). The heavy chain amino acid sequences also represent different PCR amplicons but with identical CDRs; the scFv-3 heavy chain could have been amplified using primers 8, 9, or 15, highlighted in green (Fig 7B). The scFv-7 heavy chain could have been amplified using primers 8, 9, or 10, highlighted in green (Fig 7B). (D). RT-PCR was applied using primers directed to the divergent scFv-3 and scFv-7 light chains (from the divergent CDR1 sequences (in red) to Framework 4) expressing from the NCD1.2 hybridoma cell line to establish that the light chain mRNA for scFv-3 and scFv-7 are both expressed to similar levels, as defined by the amount of PCR products after 15, 20, or 30 PCR cycles. The size of the CDR1-FR4 PCR product is approximately 300 bp and the 200 bp ladder is Hyperladder (Bioline). These data indicate that the hybridoma produces two light chains with an identical CDR3.

**Fig 10:**
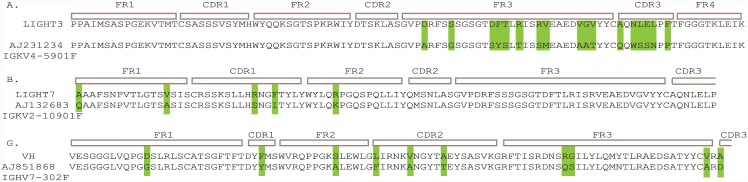
Predicted hypermutation events in scFV3 and 7. (A-C) The predicted hypermutation events that derived the scFv-3/7 (from the IMGT/V-QUEST web site).

The DNA encoding scFv-3 and scFv-7 were sequenced to define areas of presumed similarity since they both bind to the CD20 peptide. Interestingly, the heavy chains had an identical sequence; however, the light chains were different but exhibited identity in CDR3 (Fig 9A–9C). The identity of CDR3 in the light chains (AQNLELPFT) means that we cannot rule out a contribution in antigen contact by the light chain. The divergence in light chain sequence is not due to a cloning error; directed PCR to both light chain sequences using RT-PCR from RNA isolated from NCD1.2 hybridoma produces PCR products for both light chains (Fig 9D). To analyze the expression of scFV-3 light chain and scFv-7 light chain from NCD1.2 hybridoma RNA (Fig 9D), the primers used were: for the CDR1 of scFv-3 (forward primer):5’-TCTGCATCTCCAGGGGAGAA-3’; for the CDR1 of scFv-7 (forward primer): 5’-CTCCTACATAGGAATGGC-3’; and for both FR4 of scFv (3+7 reverse primer): 5’-TGATTTCCAGCTTGGTCC-3’. The predicted hypermutation events that derived the scFv-3/7 light and heavy chains are highlighted in Fig 10A–10C.

#### Cloning of recombinant scFv into synthetic biologics

**Fig 11:**
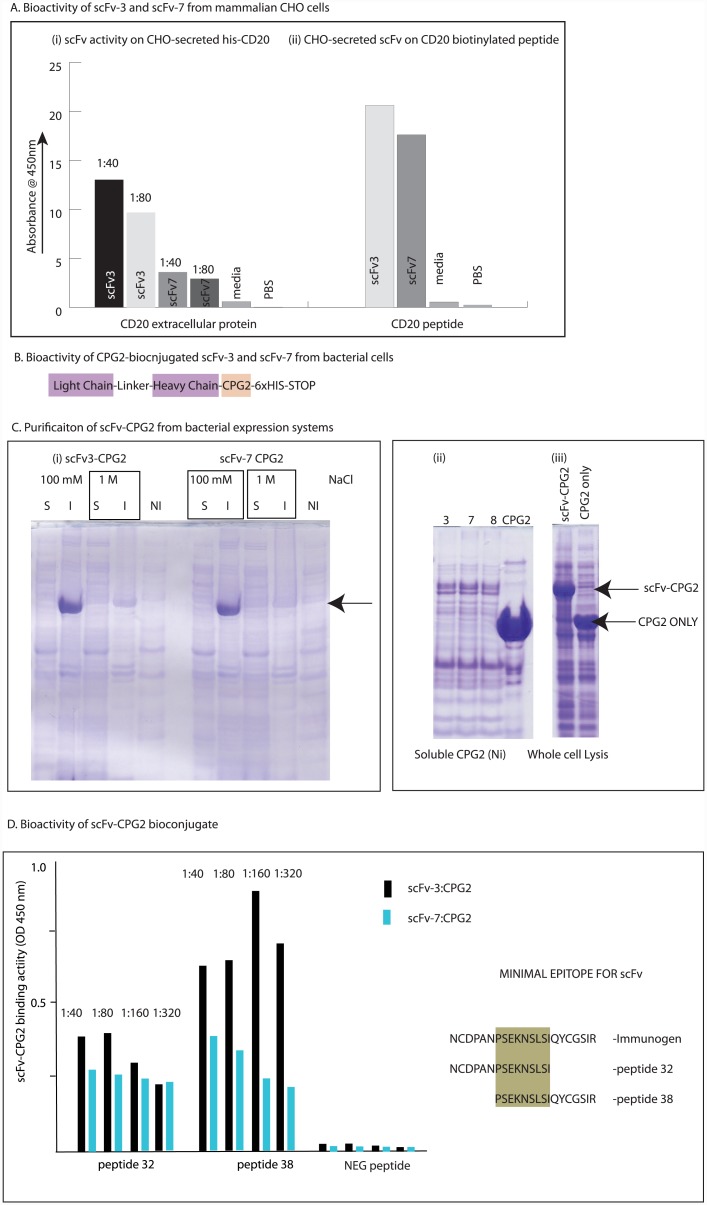
Bioactivity of recombinant scFv-3 and scFv-7 secreted from CHO cells and produced in bacteria as a CPG2-bioconjugate. (A) The indicated scFv (3 or 7) was cloned into pCDNA3.1 containing a leader sequence (amino acids MGGS) for targeted secretion into the media of tissue cultured CHO cells. (i) Binding was measured on left panel after dilution of the supernatants (1:40 or 1:80) to optimize binding against His-tagged CD20 protein; (ii) right panel the optimized supernatants of scFv-3 and scFv-7 were assayed against biotinylated CD20 peptide as indicated in the materials and methods. Antibody scFv binding to antigen was detected using peroxidase conjugated protein A and resolved with TMB-based ELISA at an OD of 450nm. Controls included media only or PBS. (B). A schematic of the scFv-3 and scFv-7 CPG2 fusion protein. (C). Optimized purification of scFv-3/7 from bacteria. Bacteria were grown and scFv-3 or scFv-7 CPG2 bioconjugates were induced with IPTG. Following lysis with 100 mM or 1 M NaCl lysis buffer, the samples were separated into soluble and insoluble fractions. These fractions were mixed with SDS loading buffer, were separated on an SDS gel, and stained with Coomassie blue. The Left panel (i) displays the solubility (S) or insolubility (I) of scFv-3 and scFv-7 from bacterial expression systems in lysis buffer containing the indicated NaCl concentrations. The arrow highlights the position of the scFv-CPG2 fusion protein. The NI lane represents the amount of soluble scFv-3 recovered from the S fraction after nickel chromatography, which was negligible. The right panel (ii) shows the relative purify of the scFv-3 and scFv-7 (and scFv-8 as a control antibody) after lysis using stabilizing lysis buffer (as in Methods) and followed by nickel affinity chromatography. The relative expression and purity of CPG2 alone is shown by comparison to highlight its enhanced yield relative to the scFv-CPG2 fusion. The insolubility remains a problem from bacterial expression systems, as the total synthesis of scFv-CPG2 and CPG2 alone is relatively similar using whole cell lysis buffer (right panel (iii). (D). Bioactivity of affinity purified scFv-3 and scFv-7 from bacteria. The normalized affinity-purified scFv fractions were assayed for binding to the indicated canine CD20 peptides (peptide 32 and peptide 38), where the core epitope resides (in gold shade). scFv-3:CPG2 was more active than scFv-7:CPG2 on both peptide 32 and peptide 38. The enhanced binding of scFv-3:CPG2 as a bioconjugate is consistent with its enhanced binding when secreted as a single chain from CHO cells and when fused to M13 gIII protein.

A key aim of this project was to determine whether this scFv targeting CD20 could be used by the comparative medicine community as a recombinant tool in the development of diverse immunotherapeutic models in canine or human disease. As such, we cloned the gene into a set of different expression vectors to determine whether it could be manipulated in principle for future applications. The scFv-3 and scFv-7 were active when fused to the gIII M13 coat protein (Fig 8C). When the scFv’s were cloned into a mammalian expression vector that directs its secretion from CHO cells, then both scFv-3 and scFv-7 supernatants were shown to produce active recombinant antibody using either CD20 protein (Fig 11A, left panel) or CD20 peptide (Fig 11A, right panel). This indicates that the CHO system can be used in principle to direct the production of recombinant protein for diagnostics, or therapeutics, in the future.

Bacterial production was evaluated after creating a CPG2-bioconjugate with an aim to determine whether the scFv scaffolds would be suitable for ADEPT (antibody directed enhanced pro-drug therapy) type immunotherapeutics[48] CPG2 is Carboxypeptidase G2, originally derived from *Pseudomonas that* has been used to convert Nitrogen mustards into potent DNA cross-linking agents[49] CPG2 was cloned into pHISTEV to create the holding vector pJGS101; in turn the scFv-3 and scFv-7 were subcloned from pCOMB3xSS into pJGS101 to create the scFv-CPG2 fusion (Fig 11B). Both of the anti-CD20 scFv-CPG2 bioconjugates were relatively insoluble in bacteria (Fig 11C, left panel). However, we optimized the production of soluble scFv-CPG2 fusion proteins that permitted affinity purification of the protein (Fig 11C, right panel). The purified scFv-3:CPG2 and scFv-7:CPG2 fusion proteins were bioactive on the CD20 derived peptides, with a proposed epitope defined by the overlapping binding towards peptide 32 and peptide 38 derived from CD20 protein (Fig 11D). These latter data suggest that although the CPG2-scFv fusions have the potential to be used as a bioconjugate, bacterial expression systems might not be the optimized source of protein for future trials in canine disease. CHO cells might remain the standard source of recombinant bioconjugated scFv-3 and scFv-7 in the future. This lower activity from bacterial systems may be due to the presumed poor protein folding pathways in bacterial systems due to very rapid translation compared to the slower protein translation rates of eukaryotic cells. For example, it has been documented that lowering bacterial translation rates can improve the folding of eukaryotic proteins[50].

## Discussion

Monoclonal antibody methodologies provide an approach to exploit the natural defense systems in mammals towards targeted or smart therapeutics. Perhaps the most successful example of this has been the wide application of the monoclonal antibody Rituximab towards a growing range of disease indications. Rituximab is a chimeric monoclonal antibody with humanized constant chains fused recombinantly to murine variable domains[51]. The success of this biologic has inspired the development of two further anti-CD20 agents for use in human therapy; tositumomab[52] and ibritumomab[53]; which are full-length mouse monoclonal antibodies that are conjugated to a radiochemical carrier. As Rituximab does not appear to bind to the canine CD20 protein, there is a need for monoclonal antibodies that can bind to the canine version of the molecule for use in comparative medicine[54]. The emergence of “companion therapeutics” has also led to the development of the first approved anti-CD20 monoclonal for use in canine lymphoma[20] and additional mouse monoclonal antibodies are being generated to CD20 thus creating an emerging competitive landscape[38].

The enhanced potential of monoclonal antibodies is derived from the application of recombinant or synthetic biology that can drive innovations in the type of bioconjugate suited to a particular platform; whether it be engaging the immune system via ADCC through use of full-length antibodies with functional Fc domains, the conjugation of radiochemical to deliver targeted radiation or toxins, or the use of recombinant nanobodies that are amenable to selected manipulation. In our approach to develop recombinant scaffolds towards canine and human CD20 protein, we have (i) screened hybridomas from mice immunized with the extracellular portion of the canine CD20 epitope for monoclonal antibodies that cross bind the human epitope for potential future inter-species application; (ii) ensured the mouse monoclonal antibody can bind CD20 in canine tissue; (iii) cloned the minimal heavy and light variable regions from mouse hybridoma cells into phage display vectors to determine whether single chain antibody domain fusions can be packaged in bioactive form; and (iv) demonstrated that a core scFv scaffold cloned from total hybridoma mRNA can be selected and fused into an ADEPT-type bioconjugate or can be secreted in active form from CHO cells. Other approaches could have been used to isolate recombinant antibodies to CD20. For instance, instead of acquiring antibodies by first producing hybridomas from CD20 immunized mice, we could have created a scFv-phage library from CD20 immunized mice. The use of a scFv library from immunized animals coupled to screening of the library on native receptor-expressed on cells could have resulted in a much larger variety of antibodies [55] [56,57].

In cloning the heavy and light chain variable domains, we thought it important to assemble the hybridoma library into M13 bacteriophage in order to select for functional antibody fragments. The formation of a scFv is not natural and its construction might disrupt the authentic CDR conformation from a naturally selected IgG. Surprisingly, we identified two scFv from the cloning that were active in CD20 protein binding. Sequencing indicated that two of the isolated scFv have the same heavy chain but different light chain sequences (although the light chains have the same CDR3), suggesting that the predominant specificity stems from the heavy chain only. An estimation of the hypermutation events that generated both light chains and the heavy chain is shown in Fig 10. RT-PCR of the original cell line indicated that, indeed, the hybridoma cell is synthesizing both light chain genes (Fig 9), although we cannot rule out that other light or heavy chains are produced by the hybridoma. For example, recent studies have shown the possible mixed chain diversity of an antibody secreting hybridoma cell; in analysing heavy and light chain transcripts from a hybridoma secreting antibodies to the transferrin receptor, three heavy-chain and three kappa (κ)-chain transcripts were identified-of which two light chains and one heavy chain were functional [58]. Bioinformatic analysis suggested that one light chain was derived from the myeloma partner of the hybridoma and the other light chain was derived from the B-cell^56^. These data indicate the coexistence of two functional κ-chain transcripts and highlights the possible complexity of the parental IgG we describe in this report. As the vast majority of antibody developments in the past twenty years have been with phage antibody libraries, the extent to which hybridomas produce heterogeneous heavy or light variable chain populations is not evident. For example, we do not know if there are additional mRNA species encoding other heavy or light chain transcripts from the NCD1.2 hybridoma. Nor do we know if the NCD1.2 IgG as secreted from the hybridoma is composed of mixed populations of two light chain encoded polypeptides. Nevertheless, of the two light chains, the scFv-3 exhibited a higher binding avidity than scFv-7 in four formats; (i) in bacteriophage as a *gIII* fusion; (ii) as a single domain protein from periplasm of bacteria; (iii) as a scFv with a leader sequence secreted from mammalian CHO cells; and (iv) as a CPG2 bioconjugate fusion protein from bacteria. We are currently cloning the single domains of the scFv-3/7 antibodies to determine whether these can be manipulated and mutagenized to further improve avidity and prospects for full caninization. In addition, we are setting up protocols for sequencing *de novo* the parental antibody IgG to determine the extent of heterogeneity of IgG sequence from the NCD1.2 hybridoma. Additionally, due to the perhaps unexpected complexity of antibody producing genes in a hybridoma cell, an alternative strategy to create a scFv-phage library form immunized mice might be better to increase diversity of active antibodies.

In summary, inter-disciplinary consortia are being developed to begin to develop complex immunodiagnostic and immunotherapeutic models bridging canine and human disease. Our 1^st^ line target for proof of concept was CD20, which has been shown to be the target of successful monoclonal antibody therapeutics for human cancer and other immunological diseases. The ability to clone the scFv directed towards canine and human CD20 offers a scaffold for the scientific community to begin to manipulate this biologic towards more innovative bioconjugate models of mammalian disease.
